# Neural Correlates of Musical Creativity: Differences between High and Low Creative Subjects

**DOI:** 10.1371/journal.pone.0075427

**Published:** 2013-09-12

**Authors:** Mirta F. Villarreal, Daniel Cerquetti, Silvina Caruso, Violeta Schwarcz López Aranguren, Eliana Roldán Gerschcovich, Ana Lucía Frega, Ramón C. Leiguarda

**Affiliations:** 1 Departamento de Neurología Cognitiva, Fundación contra la Lucha de las Enfermedades Neurológicas de la Infancia (FLENI), Buenos Aires, Argentina; 2 Consejo Nacional de Investigaciones Científicas y Técnicas, CONICET, Buenos Aires, Argentina; 3 Departamento de Física, Facultad de Ciencias Exactas y Naturales, Universidad de Buenos Aires, Buenos Aires, Argentina; 4 Facultad de Medicina, Universidad del Salvador, Buenos Aires, Argentina; 5 Departamento de Humanidades y Ciencias Sociales, Universidad CAECE, Buenos Aires, Argentina; 6 National Academy of Education, Buenos Aires, Argentina; University Medical Center Groningen UMCG, Netherlands

## Abstract

Previous studies of musical creativity suggest that this process involves multi-regional intra and interhemispheric interactions, particularly in the prefrontal cortex. However, the activity of the prefrontal cortex and that of the parieto-temporal regions, seems to depend on the domains of creativity that are evaluated and the task that is performed. In the field of music, only few studies have investigated the brain process of a creative task and none of them have investigated the effect of the level of creativity on the recruit networks. In this work we used magnetic resonance imaging to explore these issues by comparing the brain activities of subjects with higher creative abilities to those with lesser abilities, while the subjects improvised on different rhythmic fragments. We evaluated the products the subjects created during the fMRI scan using two musical parameters: fluidity and flexibility, and classified the subjects according to their punctuation. We examined the relation between brain activity and creativity level. Subjects with higher abilities generated their own creations based on modifications of the original rhythm with little adhesion to it. They showed activation in prefrontal regions of both hemispheres and the right insula. Subjects with lower abilities made only partial changes to the original musical patterns. In these subjects, activation was only observed in left unimodal areas. We demonstrated that the activations of prefrontal and paralimbic areas, such as the insula, are related to creativity level, which is related to a widespread integration of networks that are mainly associated with cognitive, motivational and emotional processes.

## Introduction

Over the last two decades, a great amount of work has been devoted to exploring the neural basis of creativity using functional neuroimaging. However, the neural networks involved in creativity, and a precise definition of it, remain elusive. There are at least three basic considerations that may explain the discrepancies between studies that currently exist. First, the concept of creativity is domain specific, i.e., mathematicians have a very different definition of creativity than do musicians. The concept is even subdomain specific, e.g., musicians differ from plastic artists, and poets seem to differ from novelists in their thought patterns and cognitive domains [1]. Second, there are no universally accepted creativity tests, and the majority of functional neuroimaging studies that have explored creativity have used very different methods of assessment [2]. Third, when assessed for creative capacities, individuals who have been professionally trained individuals in a particular field show clear differences in brain activity compared to amateur subjects [3].

Nevertheless, considering that appeal (i.e., uniqueness, originality) and appropriateness (i.e., utility, usefulness) are both defining characteristics of creativity several common findings can be extracted from most previous neuroimaging studies. Creative activity is not localized in one cerebral hemisphere; rather, it involves the integration and coordination of the process of both hemispheres. Activation of the frontal lobes (particularly the prefrontal cortex - PFC), has consistently been observed across previous studies. The PFC is a crucial structure that subserves divergent thinking, which is a critical element of creative innovation [4]. Moreover, activation of different regions of the parieto-temporal unimodal and heteromodal areas, which have extensive connections with the frontal lobes, have been found in most studies and depend on the domain evaluated and the task employed.

At present, only two studies have examined the neural substrates that give rise to the production of novel musical material. Both of these studies used the musical improvisation of professional musicians as a model. Improvisation is a prototypical creative behavior that involves free generation of melodic, harmonic and/or rhythmic musical elements that must to be adapted to the ongoing performance, must be properly monitored through auditory and somatosensory feedback and adapted to meet overall aesthetics goals [5].

Bengtsson et al. [6] used fMRI to investigate which cortical regions are involved in the generation of new musical material during improvisation on the basis of a visually displayed melody in professional classical pianists. These authors found activation in the dorsolateral prefrontal cortex (dlPFC), the pre-supplementary motor area (pre-SMA), the premotor cortex (PMC) and the posterior part of the superior temporal gyrus (STG). In turn, Limb and Braun [7] obtained different results by examining musical improvisation in professional jazz pianists using fMRI. The comparison of improvisation with the production of a well-learned musical sequence revealed deactivation of the dlPFC and lateral orbital regions and activation of the frontopolar cortex. These findings seem to be directly opposed results, although comparison of the studies is difficult because the experiments and the type of musicians evaluated were quite different.

To learn more about the possible brain correlates of musical creativity we adopted an approach that is different from those of previous studies. We investigated the brain networks involved during rhythms improvisation by comparing the brain activities of subjects with higher creative abilities to those with lesser abilities. We evaluated students of a musical school, all of which had similar levels of musical education, rather than professional musicians. The subjects were divided into two groups: a higher and lower creative group, according to the results of a creativity test (the SCAMPER, see below). We looked for differences in brain activity between the groups while participants improvised based on rhythmic musical fragments that were aurally presented. We studied improvisation because we were looking for the brain areas involved in the spontaneous generation of ideas. During improvisation, creators can develop increased spontaneity and fluency. The ideas are created without premeditation, which frees the creative process from the constraints of performing pre-composed works as evident during interpretation.

We used a minimally complex rhythm task that was devoid of melodic elements for the following reasons: i) we assumed that differences in brain activation between the higher and lower creative subjects would be easier to decipher when examined with a simpler tasks [8,9]; and ii) because rhythmic processing is clearly disassociated from melodic processing [10], the active areas observed in the higher creative subjects that were similar to those described in previous studies on creativity, could be definitively associated with the creative process, independently of the task employed.

Additionally, we used a repeat or reproduce condition to control for working memory, motor output and sensory feedback. Based on the existing literature on creativity [6,7,11] we hypothesized that we would find more activation in brain regions involved in cognitive flexibility (i.e., the prefrontal cortex) and motivation (i.e., the insula) in the higher creative group compared to the lower creative group during improvisation.

## Material and Methods

### 1.1: Subjects

Twenty four right-handed participants (mean age 21.9 ± 1.6 years; 15 females) with no history of neurological illness were recruited for this study. Subjects were selected from the Music Therapy College of the El Salvador University (Buenos Aires, Argentina) and all participants were chosen from the same degree program to reduce variation in level of musical education. All participants gave written informed consent in accordance with the declaration of Helsinki, which was approved by the Institutional Review Board of the Institute for Neurological Research-FLENI. The study protocol was also approved by the Biomedical Research Ethics Committee of the Institute for Neurological Research-FLENI

### 1.2: MRI setup

Magnetic resonance images were acquired with a 3T General Electric HDx scanner with an 8 channel head coil. Changes in blood-oxygenation-level-dependent T2* signal were measured using a gradient echo-planar imaging (EPI) sequence. Twenty four contiguous slices were taken in the AC-PC plane (TR: 2.3 s, TE: 35 ms, flip angle: 90°, FOV: 24 cm, 64 x 64 pixels per inch matrix, voxel size = 3.75 x 3.75 x 4 mm). A structural MRI was acquired with a fast SPGR-IR sequence (120 slices, 1.6-mm thick slices, TR 12.956 ms, TE 6.1 ms, flip angle 15°, FOV 24 cm, 512 x 512 matrix). One session of 150 volumes was acquired for each subject, and additional session of 50 volumes was used for habituation and practice.

Subjects lay supine in the MRI room, which was equipped with headphones, a response box place near their right hand and a mirror mounted on the head coil. The paradigm was executed using the Presentation (R) software via computer outside the scanner room.

### 1.3: Experimental design

Over the course of the paradigm, 14 different short rhythms were presented via the headphones, and each was followed by two possible task conditions: Create or Repeat. In the first case, participants were instructed to create a new rhythm based on the one they had just listened to, and in the second case, they were instructed to reproduce the rhythm they had just heard. Seven of the fourteen rhythms were used for the Create condition and the other 7 were used for the Repeat condition. The assignment of the rhythms to task conditions was counterbalanced across subjects.

The rhythms were composed of six to twelve percussive sounds played over 4 seconds. All rhythms were created with the Guitar Pro 5.0 software (the scores of the complete set are shown in Figure S1a, and the wav files of each rhythm is available in the File S1). The instrument chosen was a synthetic sound with a timbre that was similar to that of cymbal, and this sound was used exclusively throughout the study. Participants performed each task using a response box with a single button that played the role of a percussion instrument. Auditory feedback in the form of the sound of the “instrument” that was used to create the stimulus rhythms was provided through the headphones each time the button was pressed. The task duration was set at 10 seconds to give the subject sufficient time for inspiration. The instruction given was to produce two creations during this time, based on the original. In the Repeat condition subjects were instructed to replay the rhythm continuously for 10 seconds. Tasks were announced via a single word that was displayed on the screen (LISTEN, CREATE, REPEAT) for 1 second, and a blank picture following the instruction indicated that the subject should initiate performance of the instructed task. Rest periods of 5 seconds were denoted by a fixation cross that was displayed at the end of each task. There were in total 14 trials of baseline periods. In summary, the timeline of each block was as follows: listen instruction (1 s) -> rhythm presentation (5 s) -> task instruction (1 s) -> task execution (10 s) -> rest period (5 s). Before the experiments, the participants performed an extra scan session that was not included in the analysis, for the purpose of adjusting the equipment (e.g., sound, feedback) for each participant and familiarizing them with the environment. The design of the practice session was similar to those of the experiment but only the Repeat task was included and we used 5 extra rhythms that were different from those of the main experiment (Figure S1b, File S1). The total fMRI session lasted 420 seconds.

### 1.4: Performance analysis

All subjects’ performances during the fMRI session (creations and repetitions) were recorded in a text file that contained timestamps of the subjects’ button presses. For each trial, we extracted the timetable and converted it to a rhythm sequence using custom-made software running in Matlab 7.5. To create this rhythm sequence, we calculated the interval in seconds between two consecutives sounds and assigned the intervals to the corresponding notes as follows: intervals between 0.75-1.5 sec. as quarter notes; 0.37-0.74 sec. as eighth notes; 0.21-0.36 as sixteenth notes; and 0.1-0.2 as thirty-second notes. The ranges were taken as the duration of the ideal figure + 50% of its value and -50% of the neighboring shorter figure (i.e. the range of the eighth note of 0.37-0.74 sec was determined as the ideal eighth (0.5sec) + 50% of 0.5 and -50% of the ideal sixteenth (0.25)).

The creations were then evaluated by two of the authors (both musicians) using the SCAMPER technique. SCAMPER is an acronym for “substitute, combine, adapt, modify, put to other uses, eliminate and reverse”. SCAMPER is normally used to guide people in improving something that has previously be done, by posing questions to them such as, “what can I substitute?” and “What may I do instead?” This technique can be applied to any number of objects or situations and is mainly used in marketing domains [12]. In this study, SCAMPER was not used to prompt the subjects but to measure the creative product.

The quantification procedure considered two aspects of sub-classification based on Guilford’s [13] postulate. Guilford postulated that creative talent could be assessed with different variables, such as ideational fluidity and flexibility of the mind. The former measures the number of variations from the original sequence, and the latter measures the type of variation, i.e., whether the changes are substitutions, combinations, adaptations, modifications, etc.

Flexibility is accounted for by the SCAMPER items as follows: i) substitution, when a set of notes are changed for an equivalent set (e.g.,. one quarter for two eighths); ii) combination, when the order of the notes is changed; iii) adaptation, when the duration of the compass is modified for another metric; iv) modification, when new elements are inserted into the sequence via the implementation of items i or iii; v) elimination, the elimination of a beat; and vi) reversal, switching of the order of the entire sequence. For every creation we assigned one point for each type of change observed, and then we summed these points to obtain a single score. The final number assigned to each subject was the mean of that subject’s rhythm scores.

The quantification of fluidity was based only on the number of different beats generated within a creation compared to the number of beats in the original fragment. Thus, we only considered eliminations and insertions. As the fragments had duration of 4-5 seconds, and the subjects had 10 seconds to construct a creation, we defined each 10 second subject-produced sample as two creations based on the same template. Thus, the number of beats in the original fragment was doubled (see the example in the Results section for further detail). Again, the final number assigned to each subject was the mean of that subject’s rhythm scores.

Next, we used the mean fluidity and flexibility scores, to rank subjects according to their performances on both measures.

Additionally we obtained the subjects’ scores from an annual course at their university that evaluated improvisation techniques, individual creativity and musical skills. This parameter was termed ‘Cscore’.

### 1.5: Functional MRI Data analysis

Image processing was carried out using SPM5 (Wellcome Department of Cognitive Neurology, London, UK) implemented in MATLAB 7.5 (Mathworks Inc., Sherborn, MA, USA). Slice-timing correction was applied to each volume (TR = 2.3, TA = 2.2). The imaging time series was realigned to the first volume and spatially normalized to the Montreal Neurological Institute (MNI) reference brain [14]. The normalized volumes of 2 x 2 x 2 mm^3^ were spatially smoothed with an isotropic Gaussian kernel of 8mm at full width half-maximum [15] and high pass filtered (128Hz) during analysis.

Individual analyses were computed using a general linear model that included all conditions and corrections for head movements. Fixation-cross periods were use as baselines and not modeled in the design matrix. The effects were modeled using the canonical hemodynamic response function to create regressors of interest. The length of the events was set as the duration of each task. Our main goal was the analysis of the Create > Repeat comparison, hence we applied the appropriate linear contrasts to determined the active areas for this comparison for each individual.

Using the performance results we separated the subjects into two groups. The “less creative” group (LCG) included all subjects with lower fluidity and flexibility scores, and the “higher creative” group (HCG) included the subjects with greater scores in both parameters. We performed an ANOVA of two factors: Group and Condition, with two levels for each, HCG and LCG for Group, and create > baseline and repeat > baseline for Condition. The analysis yielded the following contrasts: Main effect of Group, Main effect of Condition, Interaction Group x Condition. This analysis provides a more accurate insight into the neural foundations of musical creativity. Specifically the interaction contrast should reveal whether brain activations reflecting creative actions are modulated by the degree of creativity. The ANOVA results were considered significant at the level of p < 0.05 corrected for multiple comparisons (FWE). However when no significant clusters where found after this correction, we tested the results lowering the threshold to the less stringent p < 0.001 uncorrected (all uses of this level are indicated in the Results). In all cases the minimum extended cluster size was set at k = 5. For specific identification of activity the coordinates of the final activations were converted from MNI to the stereotactic space of Talairach and Tournoux [16], although the coordinates in the tables are shown in their original MNI space.

Alternatively we used the first level create > repeat t contrast from all subjects in a regression analyses and used the creativity parameters as regressors. That is, we performed three independent regressions between the whole-brain activities defined by the aforementioned contrast and fluidity, flexibility and Cscores. These analyses resulted in a single brain map for each regression that showed the brain areas that were more strongly modulated by creativity level during the creation task than the repetition task. This approach allowed us to overcome the possible confounds that arise from the division of the subjects and the subsequent group comparison and allowed us to examine the variance explained by creativity level across all subjects.

## Results

### 2.1: Creativity Test

Evaluation of the fluidity parameter produced a distribution that ranged between 2.5 and 6.5 units. Using these data, we built a histogram from which we separated two groups: the first group was composed of 11 subjects with values below 4.5 (mean 3.41 ± 0.49) and the second group was composed of 13 subjects with values above 4.8 (mean 5.51 ± 0.54) (the differences between groups was significant: F = 81.67, p < 0.001).

The flexibility parameter values ranged from 8.2-11.3 units, and we separated the subjects in two additional groups based on a histogram constructed from these data: the first group was composed of 12 subjects with values below 9 (mean 8.71 ± 0.29) and the second group was composed of 12 subjects with values above 10.5 (mean 10.96 ± 0.18) (the differences between groups was significant: F = 81.67, p < 0.001).

**Figure 1:**
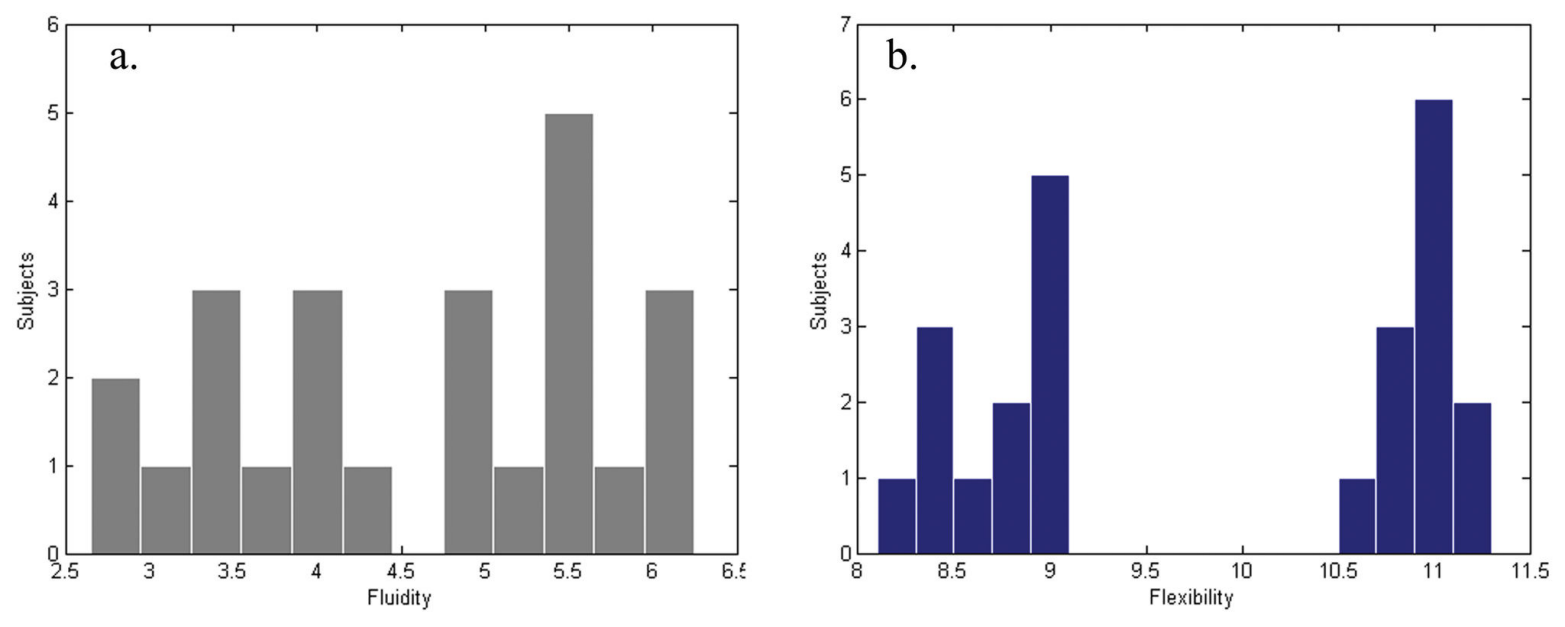
Scores distributions. Histogram of fluidity (left) and flexibility (right) scores obtained for all subjects during performance inside the scanner.

These two groupings of subjects nearly completely overlapped, i.e., the subjects in the low-fluidity group were nearly the same as those with in the low-flexibility group, with the; exception of three individuals who exhibited mixed groups assignments. Table 1 show the fluidity and flexibility values of the subjects and Figure 1 a. and b. show the respective histograms. For simplicity, we examined only two groups: the high creative group (HCG) which was formed by those subjects with high scores in both fluidity and flexibility (11 subjects) and the low creative group (LCG) which was formed by subjects (10) with lower fluidity and flexibility scores. Cscores (range 0-10) are also displayed in Table 1.

**Table 1:** Fluidity, flexibility and Cscores scores.

**Subject LCG**	**Fluidity**	**Flexibility**	**Cscores**	**Subject HCG**	**Fluidity**	**Flexibility**	**Cscores**
S6	3.43 ± 1.91	8.29 ± 2.51	5	S17	5.52 ± 0.72	10.57 ± 1.33	6
S19	2.57 ± 0.97	8.43 ± 0.95	4	S1	5.57 ± 1.06	10.83 ± 0.54	7
S4	3.29 ± 1.23	8.43 ± 0.98	4	S23	6.14 ± 0.89	10.86 ± 0.78	9
S18	3.86 ± 0.75	8.43 ± 1.28	6	S8	5.54 ± 1.12	10.87 ± 1.05	8
S11	4.86 ± 1.02	8.57 ± 0.72	7	S16	5.43 ± 1.03	11.00 ± 0.36	8
S10	4.14 ± 0.97	8.71 ± 0.81	6	S24	6.14 ± 0.54	11.01 ± 0.68	9
S7	3.57 ± 1.86	8.71 ± 1.75	4	S14	4.86 ± 0.98	11.01 ± 0.87	8
S12	5.14 ± 1.34	9.00 ± 0.56	5	S15	4.86 ± 0.87	11.05 ± 0.97	9
S20	2.71 ± 0.68	9.02 ± 1.65	5	S13	4.43 ± 0.83	11.08 ± 1.01	9
S3	3.14 ± 1.22	9.02 ± 1.85	6	S5	6.00 ± 0.33	11.09 ± 1.11	9
S22	3.29 ± 1.42	9.10 ± 1.89	6	S21	5.86 ± 0.77	11.14 ± 0.25	8
S9	4.00 ± 0.75	9.11 ± 2.32	7	S2	5.58 ± 0.65	11.42 ± 0.97	9

The subjects who were excluded from the analysis because they did not meet all group-inclusion criteria are shown in gray.

**Figure 2:**
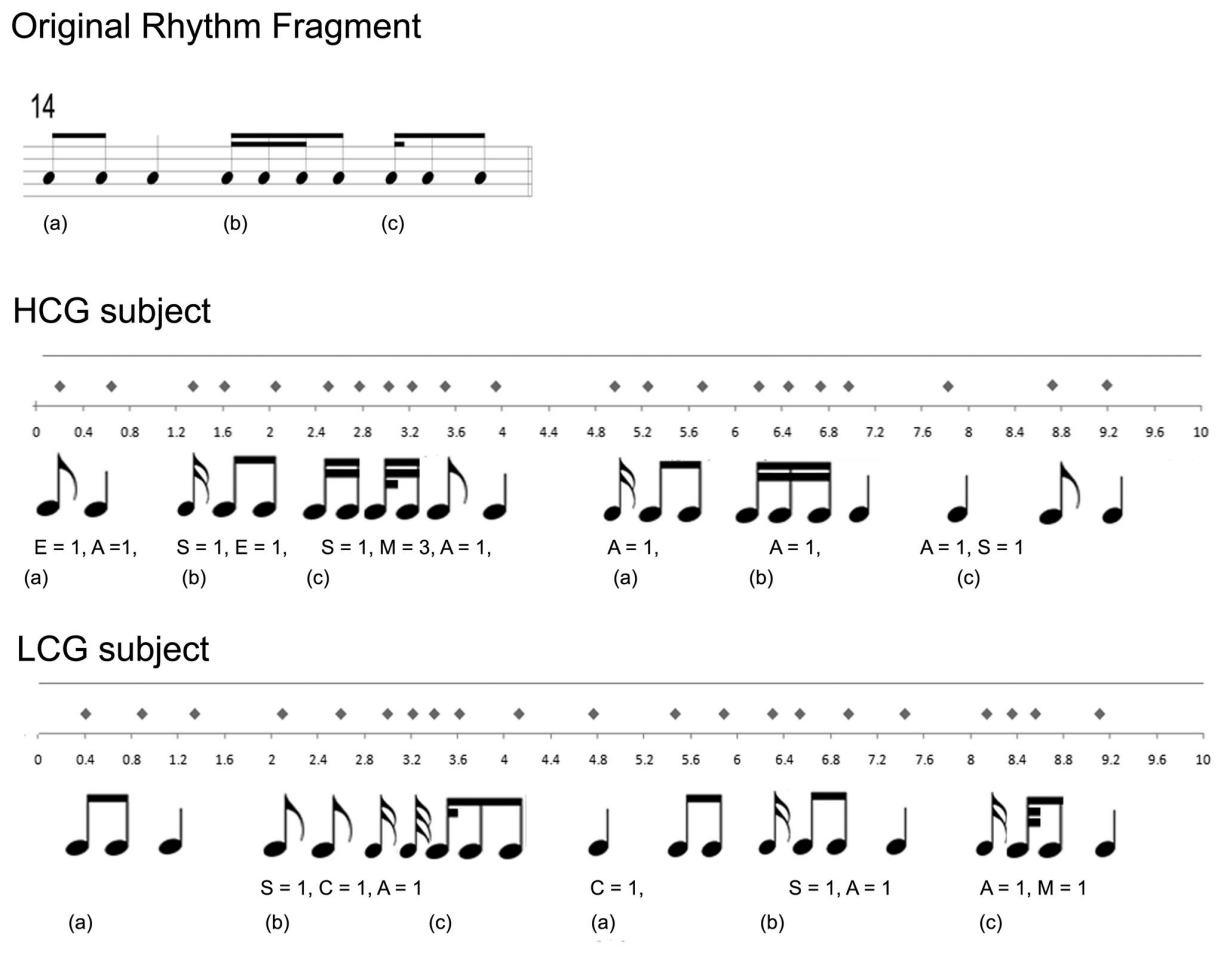
Example of a creation during the Creative Task.

Example creations from one HCG subject and one LCG subject are illustrated in musical notation in Figure 2; these examples were based on the same original fragment. The musical staff shows the original fragment. Next, for each example, we plotted the timetable registered during the scan and the transcription of the time segments in musical notation. For readers’ convenience we added labels to the template to facilitate explanation. Partial results of flexibility are shown below the figures.

 The original sequence is showed at top. One performance of a high creative subject (S2 of Table 1) is displayed in the middle and a single performance of a less creative subject (S19 of Table 1) is displayed at bottom. In both cases the timetable recorded during the scan is shown along with the transcription in musical notation and the partial punctuations of flexibility. The letters (a), (b) and (c) denote arbitrary rhythm cell segmentations used for punctuation.

Essentially, the modifications illustrated in the HCG example consisted of several substitutions, eliminations and insertions, relative to the template, i.e., in segment (a) (see Figure 2) the first two eighths were replaced by a single eighth (in SCAMPER, Adaptation (A) = 1, Elimination (E) = 1) and the next quarter was conserved (0 point). In the next rhythm cell (b), the first sixteenth was conserved but the second and third were replaced by an eighth, and the last note was not changed (Substitution (S) = 1, E = 1). The next cell (c) conserved the first sixteenth, but the next two eights were transformed into a sixteenth–thirty-second – sixteenth plus an eighth pattern, and a quarter was added at the end (S = 1, Modification (M) = 3, A = 1). At this time, a new creation that was based on the same template and the first creation began. If the second creation was identical to the first (i.e., the participant repeated the first creation) no new points were added, but if new elements were incorporated, scores for these elements were calculated. In the HCG example, after 4.8sec, the two eighths in (a) were conserved, although they were placed in other order, the last quarter was eliminated and a sixteenth was added which conserved the number of beats (C = 1, A = 1). The next three sixteenths were conserved (b), but the eighth was changed by a quarter (A = 1). Finally in (c), the metrics were changed, i.e., the last two eighths of the template were replaced by a quarter, but the number of beats was conserved (A = 1, S = 1). The total flexibility score of this example was 13.

In the LCG example, the numbers and types of changes were more limited. The first part was repeated, and in the next cell, two sixteenths were substitute for by one eighth, the order of the last eighth was changed, and a thirty-second was added (S =1, C =1, A = 1). The next cell was unchanged. The second part of the creation started with an inversion of the first cell (C = 1). In the next cell, two sixteenths were replaced by one eighth, and a quarter was added (S = 1, A =1). Finally the last cell was changed by the replacement of an eighth with a thirty-second and the addition of a quarter (A = 1, M = 1). The total score for this rhythm creation was 8.

For the fluidity parameter, we only considered the number of eliminations and insertions (E and M). Thus, the example HCG and LCG creations received scores of 5 and 1 respectively.

### 2.2: fMRI ANOVA Results

The F contrast of the main effect of Group revealed active areas within the motor cortex (precentral/postcentral gyrus) of the left hemisphere and a small cluster in the left dlPFC. The F contrast for the main effect of Conditions produced active clusters in the supplementary motor cortex (SMA), the dlPFC and the right ventrolateral prefrontal cortex (vlPFC). The Group x Condition interaction revealed activity in the left dlPFC and right insula.

**Figure 3:**
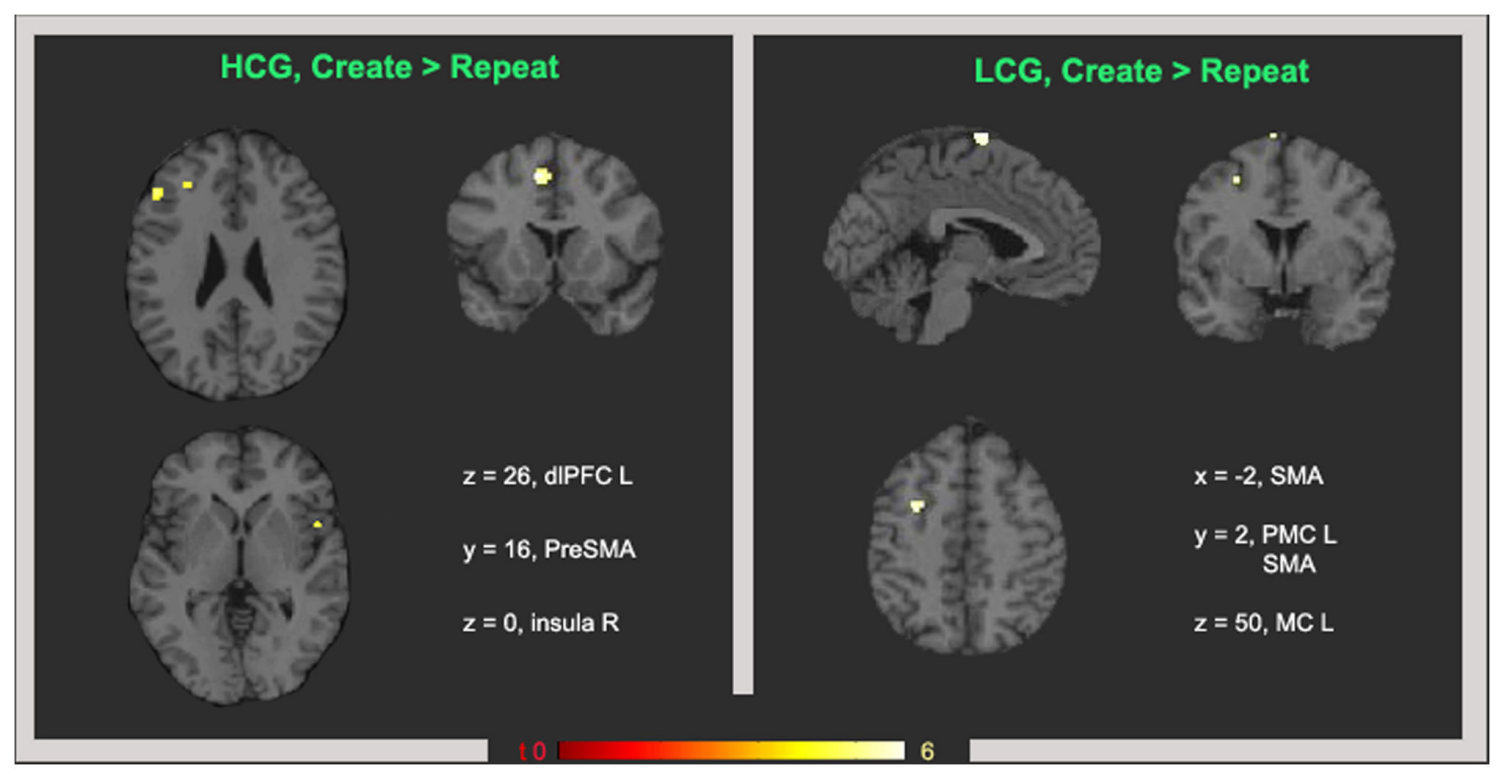
Brain activity for the Create > Repeat contrast in each group. T contrasts for the Create > Repeat comparison for the HCG (left panel) and LCG (right panel). Results are shown at p < 0.05 FWE and k = 5.

As we were particularly interested in the Create > Repeat contrast of separated group, we performed two post hoc T contrasts: HCG (Create > Repeat), and LCG (Create > Repeat). Figure 3 shows these comparisons for the HCG (left panel) and for the LCG (right panel). Activity in the left dlPFC, right insula and right vlPFC were observed for the HCG, while active clusters for this contrast were observed in the left precentral gyrus and SMA in the LCG (Table 2). The inverse comparison Repeat > Create did not produce significant activity in either group at any significance threshold.

**Table 2:** Brain activity during the Create > Repeat comparison.

**Region**	**BA**	**k**	**P corr**	***xyz***	**T**
**HCG Create > Repeat**					
LPreSMA	6	79	0.001	-8 16 50	7.1
L dlPFC	9	73	0.002	-50 32 30	6.43
R vlPFC	45	5	0.041	50 26 8	5.22
R insula	13	7	0.025	52 10 0	5.53
**LCG Create > Repeat**					
L SMA	6	72	0.002	-2-4 78	5.92
L PMC	6	16	0.0015	-28 4 50	5.5

Clusters significant at p < 0.05 (FWE).

**Figure 4:**
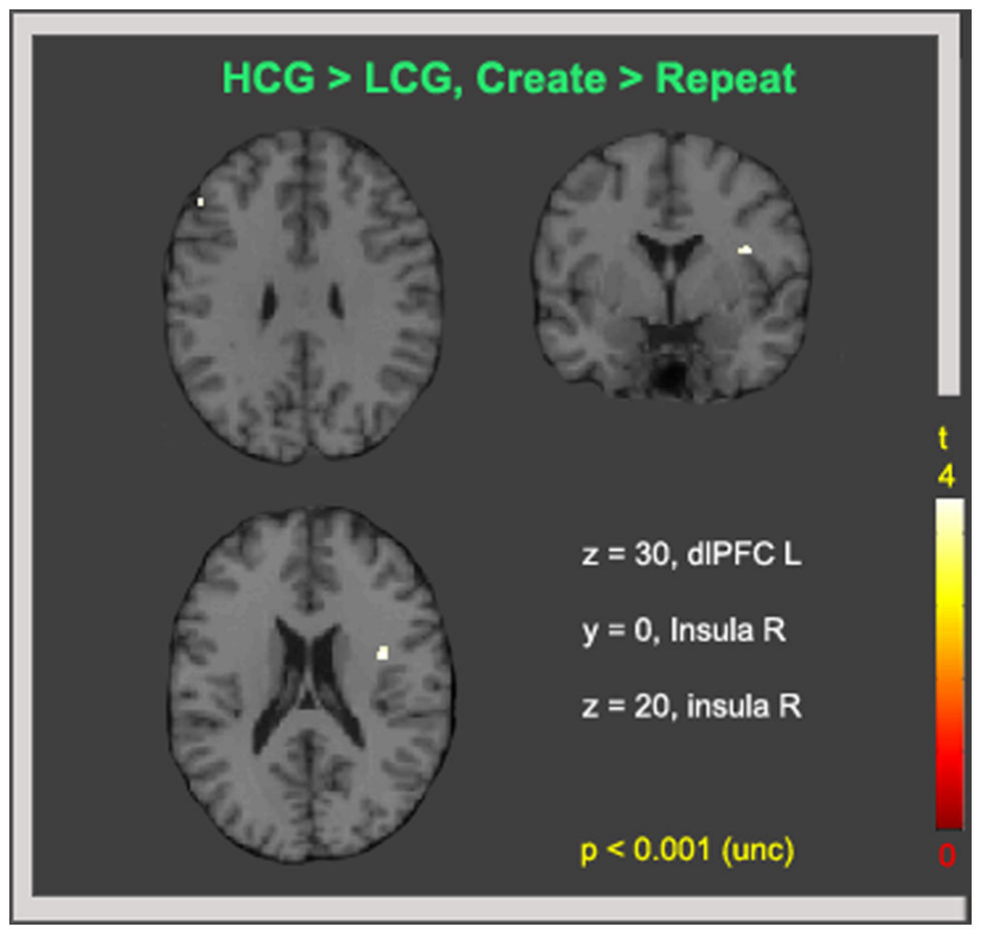
Brain activity for the Create > Repeat contrast between groups. T contrast for the [HCG (Create > Repeat) > LCG (Create > Repeat)] comparison. Results are shown at p < 0.001 uncorrected and k = 5. The inverse comparison between groups (LCG > HCG) revealed no brain activity at this threshold.

The final interaction we examined compared the latter subtractions between groups, i.e., [HCG (Create >Repeat) > LCG (Create > Repeat)] and [LCG (Create >Repeat) > HCG (Create > Repeat)]. We did not observe any significant cluster at p < 0.05 (FWE). We then examined the results using a less conservative threshold of p < 0.001 uncorrected and, obtained two frontal clusters for the HCG > LCG contrast: one in the dlPFC and the other in the right insula (Figure 4 and Table 3). Although these results had less statistical power, these active clusters were the same as the regions obtained from the HCG(Create > Repeat) contrast at p < 0.05 corrected (FWE): i.e., the left dlPFC and right insula. Specifically, the coordinate for the left dlPFC was exactly the same as the previously obtained coordinate, which reduces the possibility that this result was a false positive. The coordinate obtained for the right insula was not identical to the coordinate obtained from the previous contrast, but this coordinate was significant at p < 0.05 for the HCG (Create > Repeat) contrast when FDR rather than FWE correction was employed.

**Table 3:** Brain activity during (Create > Repeat) and HCG > LCG.

**Region**	**BA**	**k**	**P uncorr**	***xyz***	**T**
HCG > LCG					
L dlPFC	9	10	0.001	-50 32 30	3.59
R insula	13	11	0.001	38 0 20	3.69

Significant at p < 0.001 (uncorr).

The LCG > HCG contrast produce no active clusters at p < 0.001 uncorrected and produced only limited activity in dorsal PMC and paracentral lobule at the level of p = 0.003 (data not shown).

### 2.3: Regression Analyses

**Figure 5:**
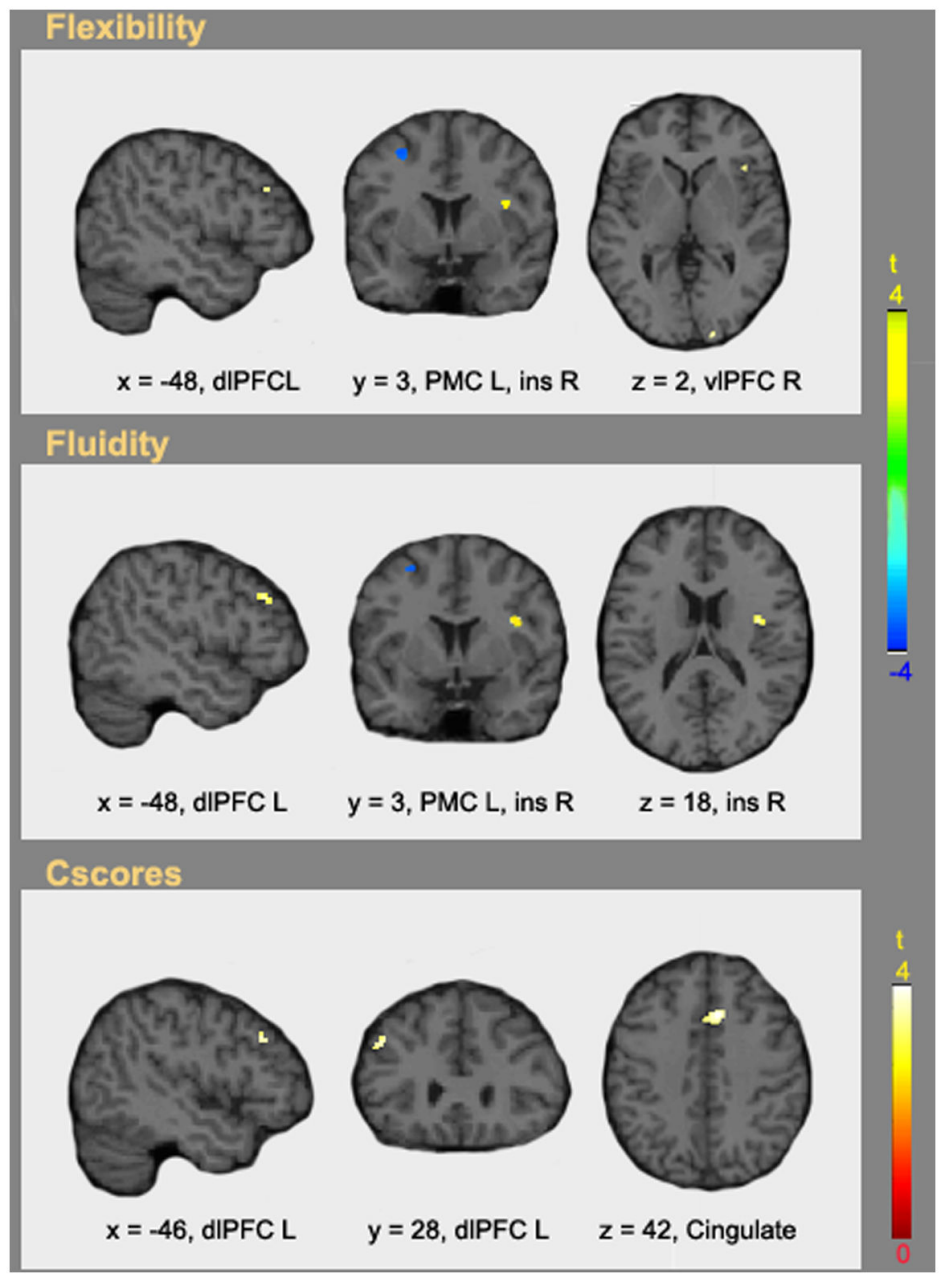
Regression analyses. Brain activity resulting from the independent regression analysis of the individuals’ Create > Repeat t contrast vs. flexibility (top panel), fluidity (middle panel) and Cscores (bottom panel). Results are shown at p < 0.001 uncorrected and k= 5.

Across the three comparisons, the regression analyses revealed significant results only at p < 0.001 uncorrected. Figure 5 shows the areas modulated by creativity level across all subjects (i.e., both groups together). Upon inclusion of the flexibility parameter as a covariate, we obtained linearly and positively modulated brain activation in the left dlPFC, the right vlPFC and the right insula. A negative correlation was observed in the PMC within the active cluster found in the LCG (Create > Repeat) contrast (Table 2). Upon inclusion of the fluidity parameter as a covariate, we obtained nearly identical results with the exception of the cluster in the right vlPFC which was not significant. Finally the external Cscore parameter was positively correlated only with the left dlPFC and the anterior cingulate gyrus. No negative correlations with Cscore were found.

## Discussion

We focused on specific characteristics of rhythmic processing, namely fluidity and flexibility, to constrain the multiple aspects of musical creativity as much as possible. When the number of rhythmic variations and the quality of such variations were considered, important and significant differences arose between highly creative and less creative subjects. First, subjects with higher level of fluidity and flexibility generated their own creations that were primarily based on modifications to the original rhythm and did not adhere strongly to the original rhythm; thus, the creations of these subjects were quite original. In contrast, the subjects with lower levels of fluidity and flexibility were adhered more strongly to the original pattern; they made whole or partial changes but always maintained references to the original stimuli. Second, we found frontal lobe activation in both hemispheres (left dlPFC, right insula and a small cluster in vlPFC) in the HCG and only found activation in the precentral and SMA regions in the LCG. Thirdly, the HCG when compared with the LCG, showed more significant increases in BOLD signal in heteromodal prefrontal region and the insula.

One salient finding of the present study was the prefrontal and right insular activation observed in the HCG, but not the LCG. Previous studies of the creative functions of the different hemispheres have produced conflicting results. Whereas some researchers, even when using non-linguistics tasks, have found more right than left hemisphere activity [17,11], many others have demonstrated different results. Thus, studies of normal subjects [18,19] and patients with commissurotomies [20] have shown, as we have demonstrated in the present investigation, that creativity is intimately dependent on, as Lezak [21] emphasized, “the bilateral integration of cerebral functions…”. However, only one of these previous studies compared highly creative subjects with less creative subjects using a measure of regional cerebral blood flow. On the basis of the Creative Functional Test, Carlsson et al. [19] selected two groups of subjects with minimal and maximal creativity. Similar to our results, these authors demonstrated that highly creative subjects show increased regional cerebral blood flow bilaterally in frontal regions when performing a divergent thinking task, whereas the less creative subjects show increases in blood flow that are predominantly restricted to the left hemisphere. Nevertheless, it is important to note, that the right frontal activation found in our HCG was minimal when examined with the FWE correction for multiple comparisons but became extensive and included left frontal activity, when tested without the correction. This is not the case in the LCG, which exhibited activation only in small frontal clusters without correction. We believe that these effects would be increased through examination of a larger sample.

Creativity is a complex, dynamic, multi-integrative process that simultaneously involves cognitive, emotional, motivational and perceptual processes [22]. The cognitive mechanisms that support creativity need to be highly flexible and include among other function, working memory, goal-directed thoughts, novelty-seeking and problem-solving which requires efficient and fluid inter- and intra-hemispheric information integration [23]. Flexibility in the generation of new ideas may be the product of unusual, distant and unexpected associations between areas involved in the detection and elaboration of novelty, particularly the PFC. Indeed, Takeuchi et al. [24] recently demonstrated a positive correlation between creativity as evaluated with a divergent thinking test, and structural connectivity between the frontal lobe and the cingulate cortex, which further emphasized the crucial role of inter- and intrahemispheric communication in creative processes. Our findings reflect this perspective because activity in regions within the PFCs of both hemispheres and the anterior insula correlated positively with the creative performance, which denotes widespread integration of networks associated with cognitive, motivational and emotional processes.

Interestingly, we found that the HCG and LCG groups showed activation of different brain regions. Because the LCG produced only partial changes to the original rhythmical patterns, the activation observed in this group mainly involved networks associated with rhythmic processing, i.e., the PMC and SMA. Moreover, it has recently been shown that an interconnected brain network integrated by neocortical (SMA and inferior parietal lobule), subcortical (i.e., the caudate) and cerebellar structures is specifically involved in complex sensory-motor integration during the performance of rhythmic movement based on auditory stimuli [25]. These findings are in line with clinical data as impaired rhythmic processing has been described in patients with parietotemporal damage [26].

The principal regions activated in the HCG were the dlPFC (BA 9) and vlPFC (BA45), pre-SMA and the insula. Additionally, when brain activation in the LCG was subtracted from that of the HCG, the dlPFC (BA 9) and the insula remained active.

Bengtsson et al. [6] have also reported activity in the dlPFC and pre SMA in professional pianists during improvisation. The authors suggested that activity in the dlPFC during creative behavior reflects the role of the dlPFC in the free selection of responses that are adapted to the overall goal of production. In addition, these authors emphasized the role of the dlPFC in selective attention and working memory during comparison of responses during improvisation with the rhythm heard and previous outputs that were aimed at avoiding repetition and regularities. Moreover, it has been demonstrated that creative people have broad focuses of attention and greater attentive capacities to properly encode and synchronize temporal events (see 1 for review). Studies of general attentive capacities comparing creative and non-creative subjects have demonstrated that subjects with fewer creative traits focus their attention more narrowly [27]. Bengtsson et al. [6] also reported activity in the premotor dorsal cortex and pre-SMA and attributed these activities to the sequential and temporal organization of behavior. This may be related to the negative correlation between premotor cortical activity and creativity level that we observed. It is possible that the main mechanism used by the LCG to produce rhythm sequences was more strongly related with to temporal organization than working memory mechanisms. However, this hypothesis requires further investigations.

Furthermore, Limb and Braun [7] found deactivation of the dlPFC and orbitofrontal cortex and selective activation of the frontopolar cortex (BA 10) during musical improvisation in a jazz pianist. These authors interpreted their findings as a reflection of defocused attention that permits the innovative, internally motivated production of fresh material without conscious monitoring. These authors also demonstrated activation in the sensorimotor idiotypic cortex and many regions of the temporal and parietal lobes. They explained the discrepancies between their findings and those reported by Bengtsson et al. [6] in terms of several important aspects, i.e., the use of different masking strategies, the study of jazz pianists who rely more heavily on improvisation than classical pianists and in the fact that they eliminated the possible impact of episodic memory encoding. The paradigm we applied is more similar to that used by Bengtsson et al [6] than that used by Limb and Braun [7]. However, the novel finding of our research is that people with high levels of creativity implement different brain mechanisms than those with low levels of creativity. Among other requirements, our HCG required a greater focus of attention, greater reliance on working memory to retain diverse musical images in their mind while others image were being processed, greater inhibition of interfering stimuli to avoid adhering to the original rhythmical patterns and greater amount of manipulating to organize their products into unique and recognizable original combinations.

The PFC receives highly processed information from all major forebrain systems [28] and represents goal-relevant information. The PFC has the ability to store abstract concepts and general principles and to learn and use rules to control behavior in a manner that allows us deal with novelty [29,30]. The capacity to break conventional and obvious patterns of thinking and adapt to new and higher order rules is central to theories of creativity [31]. The generation of new ideas based on known concepts requires the ability to select rules and maintain those rules in working memory so they can be manipulated. Dorsolateral prefrontal cortex activation has been observed during the maintenance of large sets of information and is thought to be related to the need to organize or assemble information in such a way that it can be remembered. The dlPFC is recruited as needed to manage, monitor or manipulate information that is kept active by the ventrolateral prefrontal cortex (vlPFC). The vlPFC contributes in to rule representation in a different manner. Together with the pre-SMA/SMA, the vlPFC is engaged in the maintenance of several types of abstract rules over time and potentially reflects prospective activity [32,33]. Moreover, the frontal operculum (BA44/45) has reciprocal connection with the insula. The frontal operculum has been implicated in several processes related to music, particularly processes related to the discriminative processing of pitch and rhythm [34]. For our subjects, other essential aspects in the generation of new ideas were the necessity of monitoring their production and optimization and the capacity to discriminate between internal cognitive functions, such as thought and imagination, and information that was derived from the outside world via perceptual processes (the so called reality monitoring [35]). The dlPFC and vlPFC, together with the frontopolar cortex, the insula and the lateral parietal cortex, are involved in these processes, and the prefrontal cortex in particular contributes to post retrieval monitoring operations that are required for the discrimination of thought and perception [36]. Finally, the ability to reason using relational information is central to the performance of many complex cognitive tasks. The rostrolateral PFC including parts of BA 9, 10 and 46 is important for relational integration of internally generated information, such as the comparison of retrieved relations [37]. This function is crucial for sub-goal processing in which information about one item must be maintained while another item is processed and the output of both must simultaneously be considered together: this process is crucial to creativity [38,34].

Activation of the right anterior insula, particularly the dorsal region was observed in the HCG. Recent studies have associated the anterior insula with many functions including attention, cognitive control and performance, agency, subjective feelings, emotional and motivational states and expectations (see 38 for review). Activation has been specifically demonstrated during cover singing, music listening (particularly during rhythmic tasks), listening to subjectively pleasant music and rapid sound-action associations [40,41]. Whereas the anterior insula shows activation in cognitive and social-emotional tasks, the central and posterior regions process interoceptive and sensorimotor functions respectively. However, all these functions overlap in the anterior-dorsal region which has led to the proposal that this overlap is a correlate of integration of the different functional systems [39]. Indeed, Wager and Feldman Barrett [42] concluded that the anterior insula forms a network with surrounding cortical and subcortical regions that serve to develop subjective emotional and motivational states and to translate these states into specific action plans. Thus, it is not surprising that we found a positive correlation between anterior insula activation and creativity level, which likely reflects a positive association between the capacity to integrate information and creativity level.

Creativity plays a critically important role in our everyday existence. Creativity contributes to both physical and psychological health and to optimal human functioning. Beause creativity it is a useful and effective response to evolutionary changes, its benefits are not limited to the individual but also clearly extend to society and culture. “*Creativity is the attitude by which we fulfill ourselves… It is the integration of our logical side with our intuitive side... Creativity is more than spontaneity, it is deliberation as well... It is divergent thinking, for it converges on some solution: it not only generates possibilities but also chooses among them* [43]”.

## Conclusions

Creativity requires extensive processing that involves multi-regional intra- and interhemispheric interactions. In addition to unimodal association areas that were, selectively involved due to the perceptual structures recruited by specific type of task we employed, we demonstrated activation in prefrontal and insular areas that were associated with creativity level. Working together these distant brain regions may allow an individual to trigger, modify or inhibit higher behavioral goals by maintaining internally motivated intentions, situational influences and the expected consequences of the act, thus favoring original productions. Therefore, despite the infinite nature of creative variability, our work further contributes to the delineation of the common neural organization that enables every creation.

## Supporting Information

Figure S1 **Rhythms scores.** (a) The first four musical staffs show the rhythm used in musical notation. (b) The last two staffs show the rhythms used for practice.
(TIF)

File S1 **Wav files of the rhythms played along the study: 1-14 for the experiment; 1P-5P for practice.**
(ZIP)
