# Correction: Neural Correlates of Musical Creativity: Differences between High and Low Creative Subjects

**DOI:** 10.1371/journal.pone.0094739

**Published:** 2014-04-09

**Authors:** The PLOS ONE Staff

The legend for Figure 2, “Example of a creation during the Creative Task,” appears as a paragraph below the figure in the HTML version of the paper and as the first paragraph in the left column on page 5 of the PDF version of the paper. The complete, correct Figure 2 legend is:

**Figure 2:**
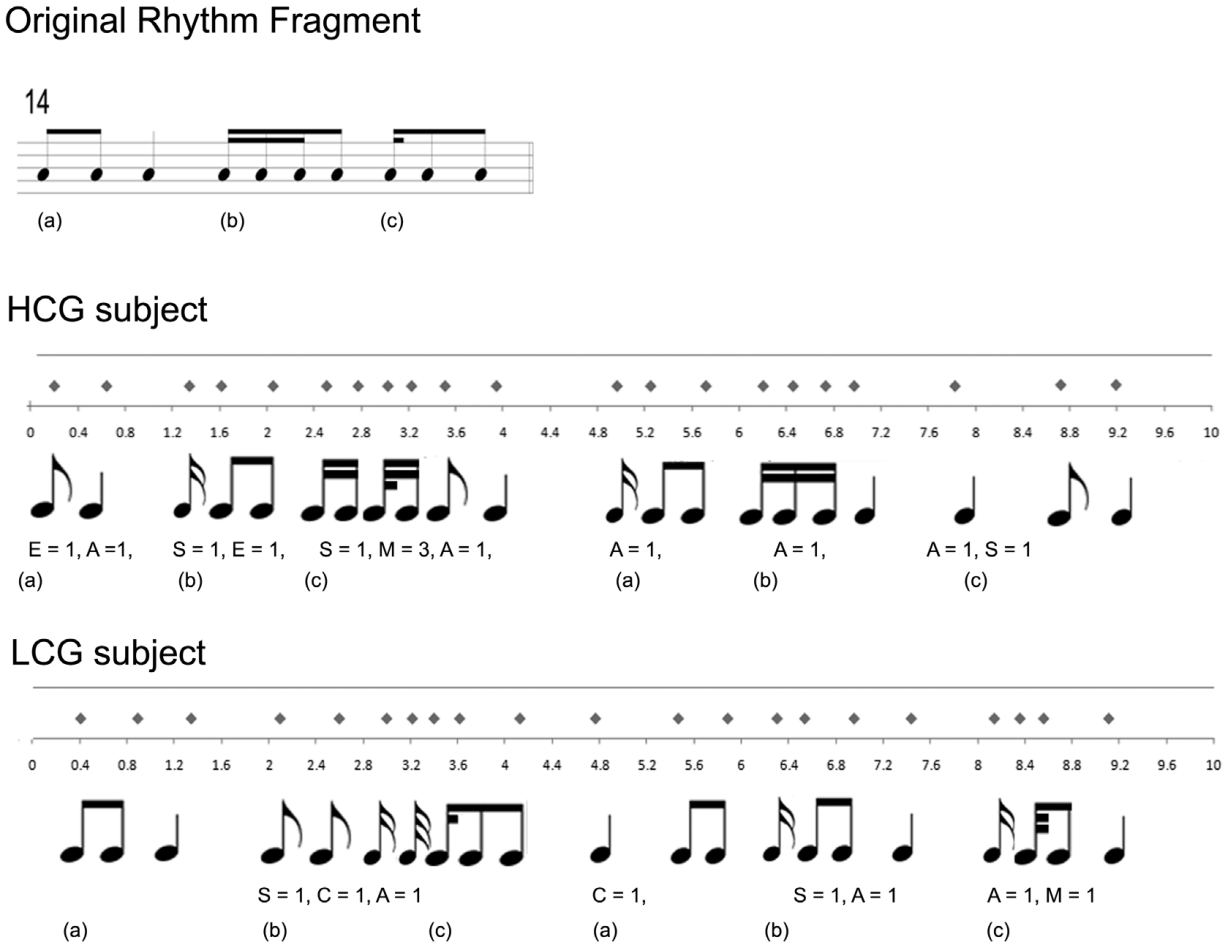
Example of a creation during the Creative Task. The original sequence is shown at the top. One performance of a high creative subject (S2 of Table 1) is displayed in the middle and a single performance of a less creative subject (S19 of Table 1) is displayed at the bottom. In both cases the timetable recorded during the scan is shown along with the transcription in musical notation and the partial punctuations of flexibility. The letters (a), (b), and (c) denote arbitrary rhythm cell segmentations used for punctuation.
