# Supplementary figures and images for: Neural Correlates of Musical Creativity: Differences between High and Low Creative Subjects
Source: PLoS One. 2013 Sep 12;8(9):e75427. doi: 10.1371/journal.pone.0075427 (PMC3771916; doi:10.1371/journal.pone.0075427)

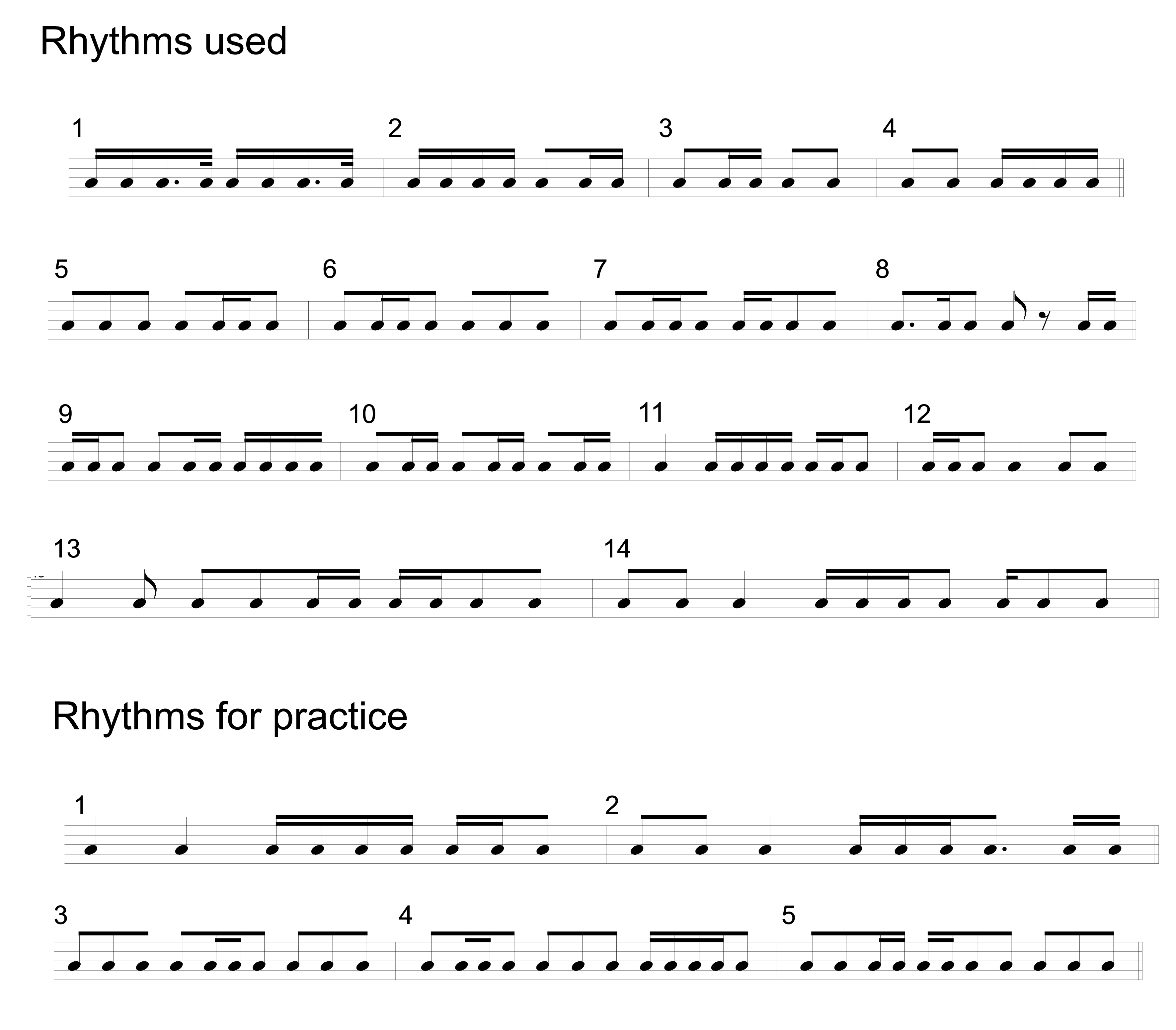

Supplement: Figure S1 — Rhythms scores. (a) The first four musical staffs show the rhythm used in musical notation. (b) The last two staffs show the rhythms used for practice. (TIF) [file pone.0075427.s001.tif]
